# Biologic TNF inhibiting agents for treatment of inflammatory rheumatic diseases: dosing patterns and related costs in Switzerland from a payers perspective

**DOI:** 10.1186/2191-1991-2-20

**Published:** 2012-09-28

**Authors:** Jan Zeidler, Thomas Mittendorf, Rüdiger Müller, Johannes von Kempis

**Affiliations:** 1 Center for Health Economics Research Hannover (CHERH), Leibniz University Hannover, Königsworther Platz 1, D-30167, Hannover, Germany; 2 herescon gmbh, Hannover, Germany; 3 Department Internal Medicine, Division of Rheumatology, Kantonsspital St. Gallen, St. Gallen, Switzerland

**Keywords:** Inflammatory rheumatic diseases, Claims data, Cost analysis, Dosing patterns, Switzerland, Tumor necrosis factor inhibitor

## Abstract

**Background:**

To obtain detailed real-life data on costs and dosing patterns in the utilisation of the TNF inhibitors adalimumab, etanercept, and infliximab in patients treated in Switzerland.

**Methods:**

Administrative claims processed by a major Swiss health insurer between 2005 and 2008 were analysed. Patients with inflammatory rheumatic diseases (IRDs) with at least one prescription for adalimumab, etanercept, or infliximab were identified. All-cause and disease-specific costs, as well as daily costs of treatment, were calculated. Dosing patterns and discontinuation rates were analysed.

**Results:**

A total of 555 IRD patients were identified. All-cause costs during the 12 months after the index event were 20,555CHF in the etanercept group, 24,152CHF in the adalimumab group, and 27,614CHF in the infliximab group. The most important cost driver was mean TNF inhibitor drug cost, which was 15,613CHF in the etanercept group, 19,166CHF in the adalimumab group, and 21,313CHF in the infliximab group. Discontinuation rates during the first year after the index event were 46.8% in etanercept, 41.3% in adalimumab, and 51.2% in the infliximab group. Rates of dosage increase were 13.3% in the etanercept group, 13.0% in the adalimumab group, and 14.1% in the infliximab group. When time on treatment was considered, daily costs of treatment were similar for etanercept and adalimumab, but were higher for infliximab.

**Conclusions:**

Marked differences in costs between subcutaneous and intravenous therapies were observed. Among the three groups of patients defined by TNF inhibitor treatment, costs for the infliximab group were highest during the year after the index event.

## Background

Inflammatory rheumatic diseases (IRDs), such as rheumatoid arthritis (RA), ankylosing spondylitis (AS), and psoriatic arthritis (PsA), are characterized by chronic inflammation of the musculoskeletal system, especially the joints and spine. The combination of disease-modifying anti-rheumatic drugs (DMARDs) and the development of tumour necrosis factor (TNF) inhibitors have for the first time been shown to induce the clinical remission of RA and delay or halt the clinical and radiological progression of the disease, thus improving the quality of life of many patients [1]. There is also clear evidence that anti-TNF therapy is efficacious in patients with AS and PsA. Accordingly, TNF inhibitors comprise an important part of current treatment recommendations [2-4].

The first available TNF inhibitors were infliximab, etanercept, and adalimumab. All three are approved for the treatment of RA, AS, and PsA. Drug costs for the TNF inhibitors used to treat IRDs are far greater than those of conventional DMARDs. Economic considerations may impact physician’s willingness to prescribe TNF inhibitors as well as the placement of these drugs in the care sequences developed by health insurance decision makers. The real-life costs of anti-TNF treatment for RA have been studied in the US and Spain; however, no data are available for AS or PsA [5-10]. Between-country differences in health care systems have resulted in a high variance in the outcomes of health economic studies and limit the generalisability of cost estimates from one country to another [11,12]. Furthermore, dosage increases in clinical practice may have significant cost implications for patients and payers, given the linear relationship between dosage and costs [5].

A study was conducted to obtain detailed real-life data on costs and dosing patterns for the TNF-inhibiting agents adalimumab, etanercept, and infliximab when used to treat IRD patients in Switzerland. Using administrative claims data from a major Swiss health insurer, we sought to estimate the all-cause and disease-specific costs of anti-TNF-treated patients, the costs incurred in different sectors of care (e.g. ambulatory care, medications, devices and aids), and the dosing patterns and discontinuation rates for TNF inhibitors.

## Methods

### Perspective

The study was designed from the perspective of the largest Swiss health insurer, Helsana, which has nearly 1.9 million insurants representing one quarter of the 7.8 million inhabitants of Switzerland. All costs of the TNF-inhibiting drugs, as well as all outpatient cost domains, were taken into account. Co-payments and out-of-pocket payments by patients are not relevant from the perspective of a health insurer. Patients in Switzerland have full coverage for most health care services with a base deductible of 300CHF and an additional co-payment of 10-20% of medication costs. The total is capped at 700CHF per year. Patients can elect to have a higher base deductible. However, due to the chronic character of IRD it is most likely that almost all of the identified patients will have selected the lowest deductible. In our study, personal contributions and co-payments by patients were not taken into account. Costs were calculated based on claims data that included co-payments made by patients. Furthermore, Switzerland has a mandatory pharmacy discount of 2.5% that is applied to drug prices, which was deducted in the analysis.

### Patients and cost domains

All patients receiving at least one prescription for adalimumab, etanercept, or infliximab during the years 2005-2008 were identified from the nationwide claims data base. Patients under the age of 18 were excluded from analysis. Some of the identified patients may have been treated with a TNF inhibitor for a condition other than an IRD (e.g., Crohn’s disease, psoriasis). Since ICD codes were not recorded in Swiss claims data following legal regulations, we elected to exclude patients who were treated in two or more quarters per year by a gastroenterologist or dermatologist.

In addition to basic claims data, which included information on age and gender, detailed information was extracted on a per-patient basis for the following cost domains: outpatient care, ambulatory care provided in hospitals, medications, laboratory tests, devices and aids, and other claims.

The cost domain “ambulatory care provided in hospitals” included health services such as medical consultations and treatments, infusions, reports by doctors, and ultrasonic testing and radiographs that were provided on an outpatient basis by hospitals. Cost data for inpatient treatment episodes were not available in the data set on a per-patient basis. The treatment date and identity of the specialist group of the treating physician were available for most claims.

Information from the different health care sectors was linked via an unique identification number for each patient. Health services that could not be included explicitly in one of the specific cost domains were summarised in the category “other claims”.

### Cost analyses

The study was designed as a cost analysis. This type of methodology provides information about the frequency of use and types of different therapies, costs of therapy, treatment patterns, and general conditions in health care for a specific disease. Annual costs were calculated for the year following the index event. The first anti-TNF claim in the study period was selected as the index claim or event. Only patients for whom data were available for at least a full year after the index event were included in the analysis. Patients were classified according to the index TNF inhibitor, i.e., etanercept, adalimumab, or infliximab. Patients who switched therapy to another TNF inhibitor within the follow-up period were not included in the analysis.

### IRD-related versus non-IRD-related direct costs

The study aimed at discriminating IRD-related from non-IRD-related direct costs. For the identification of IRD-specific resource use, standardised classifications in Swiss health care were used, including the Tarmed classification, the pharmaceutical specialties list (Spezialitätenliste), the laboratory analyses list (Analyseliste), and the “Mittel- und Gegenstände-Liste” (MiGeL). Based on these classification systems, each individual claim was classified as IRD-related or not-IRD-related for the analysis. To enhance the accuracy of this approach, some claims were classified as IRD-related only if they were provided by a rheumatologist (i.e., more general services which could also relate to other conditions if invoiced by a general practitioner). Therefore, IRD-related costs were calculated only for patients who were treated at least once by a rheumatologist during the year after the index event.

### Analysis of dosing patterns

TNF inhibitors are available in different administration forms and dosages. Etanercept and adalimumab are administrated subcutaneously (SC). The recommended dosage for etanercept is 25mg twice a week or 50mg once weekly. Adalimumab is recommended at a dosage of 40mg every other week, but the dosage can be increased to 40mg weekly. Infliximab is administrated intravenously (IV) with a recommended dose of 3mg per kg of body weight for RA and 5mg per kg for AS and PsA. Additional infusions are administered 2 and 6 weeks after the initial infusion and at 8-week intervals thereafter. In addition, the dosing can be increased and infusion intervals shortened depending on patient needs or other circumstances. Since the dosing of infliximab depends on the patientÂ’s body weight, which was not available in the database, the dose administered in the third infusion (i.e., associated with the third claim) was taken to be the recommended patient dose for the analysis. For patients who had less than three infliximab claims, the dose associated with the first claim was taken to be the recommended dose.

The time horizon was calculated for each claim, for which the prescription should suffice, assuming that the treatment was initiated with the recommended dosing scheme. Using this approach, an average annual treatment length was calculated, i.e., the average number of days for which the filled prescriptions should last assuming treatment based on labelling recommendations. For example, a prescription of one syringe of adalimumab was assumed to last for 14 days. The average annual treatment length within the respective time horizon was calculated for one, two, and three years after the index event. For the analysis of the first year only patients who had at least one year between the first and last TNF inhibitor claim were taken into account for the analysis. The same approach was used when performing the two- and three-year analyses, i.e. only patients who had at least two- and three-years between first and last TNF inhibitor claim were included.

In addition, a separate calculation of adherence patterns was conducted using the methodology proposed by Wu et al. who investigated TNF inhibitor discontinuation rates and treatment patterns in a US setting [13]. Discontinuations in treatment were defined as:

• For etanercept and adalimumab, a gap of more than 60 days between the end of the recommended dosage and the following claim.

• For infliximab, discontinuation was assumed if one of the following cases occurred:

1)the gap between the first and second infusion was more than 14 plus 60 days,

2)the gap between the second and third infusion was more than 28 plus 60 days,

3)the gap between subsequent infusions was more than 56 plus 60 days.

A prescription gap was defined as the number of days between a TNF-inhibitor prescription and the following prescription. With the first appearance of a gap, a patient was classified as being not continuously treated. In a third analysis step, the percentage of patients receiving more than the recommended dosage was calculated using the approach of Wu et al. [13]. The weekly dosage for each prescription was calculated as:

• Quantity x 7 / prescription gap (for adalimumab)

• Dosage x Quantity x 7 / prescription gap (for etanercept)

• Number of vials x 7 / prescription gap (for infliximab)

The average dosage within the first year of treatment was compared with the recommended dosage (e.g., 0.5 syringes per week for adalimumab). For infliximab, the reference dosage was chosen to be that associated with the third claim. An increase in dosage was defined as an observed average weekly dosage 33.3% higher than the recommended dosage (etanercept or adalimumab) or an average weekly dosage 33.3% higher than the reference dosage (infliximab). Patients with fewer than two claims for any TNF inhibitor were excluded from the analysis.

### Analysis of average daily costs of treatment

In addition to the annual costs of TNF inhibitor treatment, daily costs of treatment were analysed. For each patient, the number of days with active TNF inhibitor treatment was calculated taking into account the recommended dosage. All active treatment-related costs were aggregated and divided by the number of days of treatment in order to estimate the mean daily treatment cost.

### Software, statistical analyses and data protection

Data management and statistical analyses were performed using Microsoft® Access 2007 and Excel 2007. Additionally, SPSS version 15 was used for specific statistical analyses. In general, comparisons among the anti-TNF treatment cohorts were made using independent groups t tests for interval variables and chi-square test for nominal variables. Wilcoxon tests were performed to facilitate cost comparisons among the three groups. All tests were performed with two-tailed α = 0.05.

The data were available on a pseudonymised basis. Pseudonymisation precluded the identification of individual patients and de facto means anonymous data ensuring data protection demands. Hence, current data protection regulations were addressed with this approach.

## Results

### Study population

**Figure 1:**
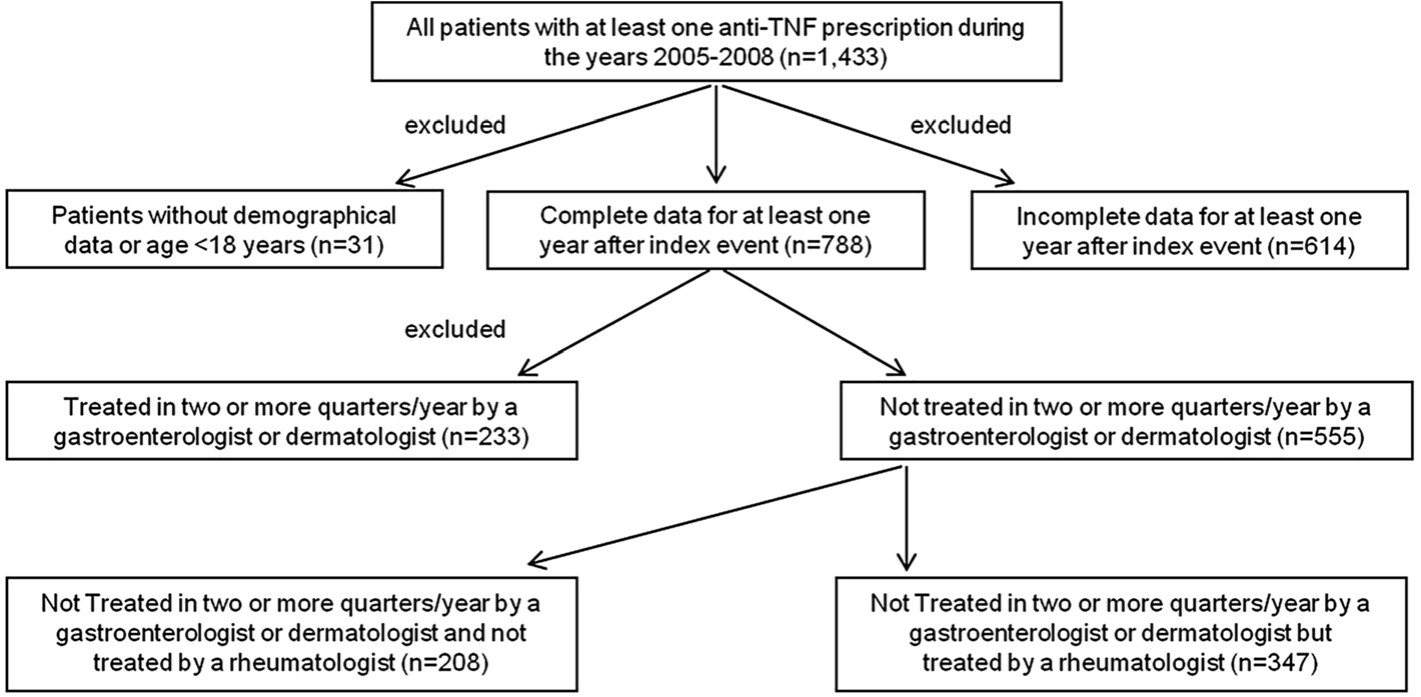
Study population.

A total of 1,433 patients with at least one anti-TNF claim between 2005 and 2008 were identified and selected. 788 of these patients were ≥18 years of age with full evaluable data for at least one year after the first anti-TNF claim. Of these patients, 555 were not treated in two or more quarters by a gastroenterologist or dermatologist and were defined as IRD patients (Figure 1).

The mean age of these patients was 50 (SD: ±15.31) years, and 59% were female. The infliximab group was younger and included a greater proportion of males than the other two groups (Table 1).

**Table 1:** Number of patients and demographic data

	**Etanercept**	**Adalimumab**	**Infliximab**
Patients	233	201	121
Mean age (standard deviation)	54 (15,20)*	51 (15,12)**	41 (12,16)
Female, %	63***	59	51

* p < 0.05 compared with adalimumab and infliximab.

** p < 0.05 compared with infliximab.

*** p < 0.05 compared with infliximab.

To ensure the accuracy of the calculation of IRD-related costs, some claims were defined as IRD-related only if the related service was provided by a rheumatologist. Therefore, IRD-related costs were calculated only for those patients who were treated by a rheumatologist. 347 of the 555 patients were treated at least once by a rheumatologist during the year after the index event. The mean age of these patients was 51 (±16.16) years, and 63% were female. 153 of these patients were treated with etanercept, 150 with adalimumab, and 44 with infliximab.

### Annual costs of treatment

Costs during the 12 months after the index event were 20,555CHF in the etanercept group, 24,152CHF in the adalimumab group, and 27,614CHF in the infliximab group (Table 2). Costs in the SC group (adalimumab and etanercept) were significantly lower than those in the IV group (infliximab) (p < 0.001). Medication costs were by far the most important cost driver. These were 17,751CHF in the etanercept group, 21,315CHF in the adalimumab group, and 22,666CHF in the infliximab group.

**Table 2:** Descriptive comparison of healthcare cost for the 12 months after the index event in CHF per patient

	**Mean (SD)**	**95%-CI**	**Minimum**	**Maximum**
**Etanercept (n = 233)**				
Outpatient care	1,113 (998)	984-1,241	0	5,775
Medications	17,751 (10,024)	16,457-19,045	1,058	102,156
Laboratory tests	585 (531)	517-654	0	4,114
Outpatient care in hospitals	453 (1,206)	297-609	0	12,382
Devices and aids	60 (220)	32-89	0	1,827
Other claims	593 (1,801)	360-825	0	19,749
*Total direct cost (etanercept*)	20,555 (10,740)	19,169-21,942	1,589	111,994
**Adalimumab (n = 201)**				
Outpatient care	1,276 (965)	1,142-1,410	0	5,798
Medications	21,315 (9,008)	20,062-22,568	1,169	58,129
Laboratory tests	634 (521)	561-706	0	4,840
Outpatient care in hospitals	418 (891)	294-542	0	5,913
Devices and aids	90 (358)	40-140	0	4,004
Other claims	419 (810)	306-531	0	7,241
*Total direct cost (adalimumab)*	24,152 (9,403)	22,844-25,460	1,994	61,613
**Infliximab (n = 121)**				
Outpatient care	1,087 (1,243)	863-1,310	0	6,358
Medications	22,666 (10,625)	20,754-24,579	4,247	68,590
Laboratory tests	1,024 (751)	889-1,159	0	4,176
Outpatient care in hospitals	2,363 (2,817)	1,856-2,871	0	23,271
Devices and aids	151 (258)	104-197	0	1,722
Other claims	323 (703)	196-449	0	4,304
*Total direct cost (infliximab)*	27,614 (11,860)	25,479-29,748	5,733	72,233

Specific IRD-related costs during the 12 months after the index event were 16,824CHF (±8,589) in the etanercept group, 20,532CHF (±9,228) in the adalimumab group, and 23,952CHF (±11,833) in the infliximab group. IRD-related costs were significantly lower in the SC group than the IV group (p = 0.014). The most important cost drivers were TNF-inhibitor drug costs with 15,613CHF (±8,368) in the etanercept group, 19,166CHF (±9,075) in the adalimumab group, and 21,313CHF (±11,557) in the infliximab group.

### Dosing patterns

**Figure 2:**
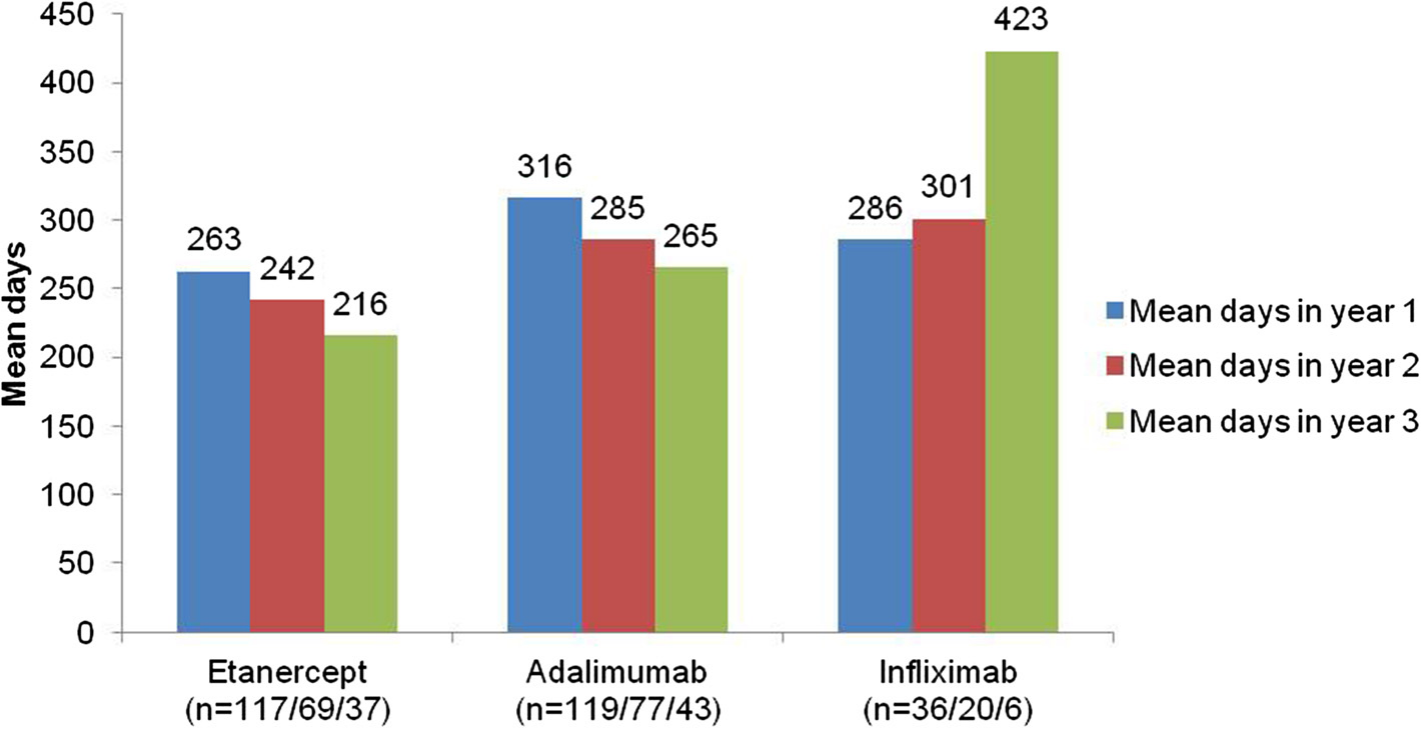
Averaged annual treatment over three years.

For patients who continued on therapy with any of the three TNF-inhibiting agents, the average annual inter-application length of therapy (etanercept or adalimumab) decreased during the two years following initiation (Figure 2). For example, patients treated with etanercept throughout the first year (n = 117) received active substance for an average of 263 days with subsequent declines over years 2 (n = 69, 242 days) and 3 (n = 37, 216 days). In contrast, the average annual treatment days in the infliximab group increased from 286 days (n = 36) in year 1 to 423 days (n = 6) in year 3.

The discontinuation rates during the first year after the index event were 41.3% in the adalimumab group, 46.8% in the etanercept group, and 51.2% in the infliximab group. There was no significant difference between the SC and IV groups (p = 0.172). The dosage increase rate was 13.0% in the adalimumab group, 13.3% in the etanercept group, and 14.1% in the infliximab group one year after index event. There was no significant difference between the SC group and IV group (p = 0.832).

### Daily costs of treatment

Patients stop taking these kinds of therapies for different reasons. Lack of efficacy and occurrence of adverse events range among the most important of the potential reasons. The cost per patient for these therapies only for the time period that patients are under active treatment with one of these products is under current discussion. This might explain the need for additional resources during that time and gives a view without the “confounder” of patients stopping treatment. There were no differences in daily treatment costs between the SC therapies (i.e., etanercept and adalimumab). However, significant differences in treatment costs were observed between the SC group and IV group (p < 0.001) (Table 3).

**Table 3:** Descriptive comparison of mean daily treatment costs per patient for active treatment during the 12 months after the index event in CHF

	**Mean (SD)**	**95%-CI**	**Minimum**	**Maximum**
**Etanercept (n = 233)**				
Outpatient care	4 (11)	3-6	0	164
Medications	83 (20)	80-86	62	301
Laboratory tests	2 (2)	2-2	0	18
Ambulatory care in hospitals	1 (3)	1-1	0	18
Devices and aids	0 (1)	0-0	0	5
Other claims	2 (7)	1-3	0	95
*Total daily cost (etanercept)*	92 (26)	89-96	69	328
**Adalimumab (n = 201)**				
Outpatient care	4 (4)	4-5	0	22
Medications	84 (12)	82-86	65	159
Laboratory tests	2 (2)	2-2	0	21
Ambulatory care in hospitals	1 (3)	1-2	0	17
Devices and aids	0 (1)	0-0	0	11
Other claims	1 (3)	1-2	0	29
*Total daily cost (adalimumab)*	93 (14)	91-95	68	169
**Infliximab (n = 121**)				
Outpatient care	4 (5)	3-4	0	25
Medications	118 (84)	103-133	36	823
Laboratory tests	3 (3)	3-4	0	17
Ambulatory care in hospitals	9 (9)	7-10	0	65
Devices and aids	1 (1)	0-1	0	4
Other claims	1 (2)	0-1	0	12
*Total daily cost (infliximab)*	135 (88)	119-151	63	878

## Discussion

A nationwide cost analysis of the outpatient anti-TNF treatment of patients with the most prevalent IRDs in Switzerland was performed using data from a large health insurer. A key strength of this study is that results were based on real incurred costs data which represents the daily life treatment setting. This approach is useful for identifying health care costs from a health insurer perspective.

There were marked differences in the descriptive comparison of costs among the etanercept, adalimumab, and infliximab groups. Etanercept had the lowest and infliximab the highest all-cause costs during the year following the index event. The relative magnitudes of these costs were consistent with the calculated IRD-related costs. Etanercept and adalimumab had similar daily costs of treatment, while infliximab daily costs of treatment were higher than both of the SC treatments. Earlier US-based studies involving only RA patients reported similar findings [5,6,14]. Studies conducted in institutional settings have also reported infliximab to be costly relative to other TNF inhibitors. For instance, a study using data collected in 2005 from RA patients treated at Spanish hospitals found that patients treated with etanercept had lower health care costs than those treated with infliximab [9]. Dissimilar from earlier investigation, we estimated costs for patients with any of several IRDs and over a more recent time horizon (i.e., 2005 through 2008). Thus, our findings suggest that cost differences between infliximab and the other TNF-inhibitors are a persistent phenomenon and continue to be relevant in the Swiss outpatient setting.

Dosage increases in clinical practice may have significant cost implications for patients and payers, given the linear relationship between dosage and costs [5]. A recently published RA treatment algorithm advocates shortening the dosing interval of adalimumab or increasing the dose or shortening the dosing interval of infliximab in patients with an inadequate response prior to switching to another TNF inhibitor [15]. Therefore, it is important to understand the dosing regimens used for TNF inhibitors in clinical practice. Dosage increase rates were moderate in all three treatment groups in the current study. However, when assessing dosing patterns over three years, an increase of the average annual treatment length was observed for infliximab in years 2 and 3 after the index event. In contrast, the number of treatment days decreased in subsequent years for etanercept and adalimumab. Upward dosage adjustment of infliximab in patients with RA has been associated with increases of 30–50% in medication costs in recent studies [6,7]. For instance, Harrison et al. reported that among naive and continuing patients, dose increases from the first to the last prescription were more likely to occur for infliximab (26% and 24%, respectively) then adalimumab (10% and 9%, respectively) or etanercept (1% and 3%, respectively) [5]. In our study, upward dose adjustments for RA and the higher approved doses for AS and PsA (i.e., 5 mg/kg vs. 3 mg/kg for RA) could have contributed to higher medication costs for infliximab. Higher costs for outpatient care in hospitals, presumably for IV administration charges, may also have influenced the observed infliximab costs.

The current study has several limitations. The observed differences in costs may have been due to differences in the proportions of RA, AS, and PsA patients in each treatment group as well as differences in disease severity and activity among the investigated IRDs. This is a limitation of retrospective claims data analyses in Switzerland where clinical data are not available. ICD codes are not recorded in Swiss claims data. This prevented us from differentiating among patients with RA, AS, or PsA. To address this problem, IRD-patients were identified from visits to rheumatologists, and patients with a visit to a gastroenterologist or dermatologist were excluded (since Crohn’s disease or psoriasis could have been the indication for the visit). The infliximab group included significantly greater proportions of males and younger patients than the other treatment groups. This suggests that the infliximab group may have included a higher proportion of AS patients, which could have magnified between-group cost differences due to the dosing used for infliximab in AS patients. Patients who died during the observation period were excluded due to the fact that only patients for whom data were available for at least a full year after the index event were included in the analysis. However, there is no reason to expect that cases were disproportionately distributed among the three study cohorts as there is no evidence to support that there are different mortality rates as a follow-up from therapy between the three TNF inhibitors. Patients who switched therapy to another TNF inhibitor within the follow-up period were not included in the analysis. This group could contain several patients who did not respond or did not tolerate the index TNF inhibitor.

A further limitation with respect to the interpretation of the results comes from the fact that incident as well as prevalent cases are included in the analyses. It was not possible to separate these two types of patients as the data set was limited. Nevertheless, this only relates to the absolute figures of the data presented as there is no reason to hypothesize that there are relative differences between the different groups regarding this point. Finally, the dosing calculations were based on filed claims paid by the health insurer. Thus, the results may not reflect the actual amount of infliximab administrated to the patient and may over- or underestimate the dosage for any infusion. For example, a patient increasing from 1.2 to 1.8 vials would have appeared to have a stable dose of 2 vials if the interval between infusions remained the same, whereas a patient increasing from 1.6 to 2.2 vials would have been considered to move from 2 to 3 vials.

In summary, the results of this study provide insights into the costs of IRD patients treated with TNF inhibitors in Switzerland and are the first real-world cost estimates for the entire spectrum of rheumatic diseases for which TNF inhibitors are approved to treat. Our findings reflect the actual medical use of TNF inhibitors without the limitations of clinical trials and allow for a head to head comparison although the inherent limitations of retrospective claims data analysis does not allow causal conclusion. Given the availability of several TNF inhibitors with comparable efficacies [16,17] but different costs, the findings of this study can assist payers in understanding the financial burden of the treating IRDs with anti-TNF agents and making meaningful cost comparisons. For generalization, further studies, which combine costs of TNF inhibitors with patients clinical characteristics (e.g., disease duration, pain severity, disease activity), which may affect both drug choice and treatment outcome, are certainly needed.

## Conclusions

This claims data study demonstrates marked differences in costs between subcutaneous and intravenous therapies for patients with inflammatory rheumatic diseases. Among the three groups of patients defined by TNF inhibitor treatment, costs for the infliximab group were highest during the year after the index event. Dosage increase rates were moderate in all of the treatment groups. No significant differences could be observed for dosage increases and discontinuation rates.

## Competing interests

The authors have declared no conflicts of interest.

## Authors’ contributions

JZ was responsible for the conception and design of the study, performed the statistical data analysis and drafted the manuscript. TM was involved in the conception and design of the study, reviewed the manuscript and revised it critically for important intellectual content. RM helped to draft the manuscript and revised it critically for important intellectual content. JvK helped to draft the manuscript and revised it critically for important intellectual content. All authors read and approved the final manuscript.
